# Bee pollen as a source of phenolic compounds in potato snacks

**DOI:** 10.1038/s41598-025-09776-4

**Published:** 2025-07-07

**Authors:** Agnieszka Nemś, Sabina Lachowicz-Wiśniewska, Ireneusz Tomasz Kapusta, Joanna Miedzianka, Agnieszka Kita, Ángel A. Carbonell-Barrachina

**Affiliations:** 1 Department of Food Storage and Technology, The Faculty of Biotechnology and Food Sciences, Wroclaw University of Environmental and Life Sciences, Chełmońskiego 37 Street, 51-630, Wrocław, Poland; 2 Department of Medicinal and Health Science, Calisia University, 4 Nowy Świat Street, Kalisz, 62-800 Poland; 3 Department of Food Technology and Human Nutrition, University of Rzeszów, 4 Zelwerowicza Street, Rzeszów, 35-601 Poland; 4 Grupo de Investigación “Calidad y Seguridad Alimentaria”, Centro de Investigación e Innovación Agroalimentaria y Agroambiental (CIAGRO- UMH), Universidad Miguel Hernández de Elche, Carretera de Beniel, km 3.2, 03312-Orihuela, Alicante, Spain

**Keywords:** Antioxidant activity, Bee products: extrusion, Pellet snacks, Phenolic profile, Total flavonoids, Analytical chemistry, Environmental sciences

## Abstract

The study examined the effects of adding bee pollen to potato snacks on their chemical composition, antioxidant properties, and sensory characteristics. Bee pollen from six different sources varied in phenolics and flavonoid content and composition, antioxidant activity and color. The four bee pollens with the highest content of bioactive compounds were used in the preparation of potato snacks obtained by frying extruded pellets with 1, 3 and 5% of bee pollen. During snack production, total phenolic and flavonoid contents decreased by 4.76–65% and 69–80%, respectively, while antioxidant activity was reduced by 9–72% depending on the used level of addition and type of bee pollen. Despite this, snacks enriched with 1–5% bee pollen exhibited significantly higher levels of phenolics (1.3–2.6 times), flavonoids (2.0–4.8 times), and antioxidant capacity (ABTS^•+^ 1.6–3.7 times, DPPH^•^ 1.1–1.4 times, FRAP 1.8–5.6 times) compared to controls. The highest antioxidant capacity was observed in snacks with 5% rapeseed and multifloral bee pollen. Sensory analysis highlighted snacks with 3% multifloral bee pollen as those with the highest quality. This research has demonstrated that bee pollen can be a promising fortification ingredient, improving the nutritional value of snacks while masking its naturally bitter taste, if used in relatively low percentages (< 5%). These findings supported the development of healthier snack options enriched with bee pollen for the functional food market.

**Supplementary Information:**

The online version contains supplementary material available at 10.1038/s41598-025-09776-4.

## Introduction

Pollen is the male reproductive cells produced by flowers^1,2^. Bee pollen is flower material collected from plant anthers and properly processed by honey bees (mix with secretion from salivary glands or nectar and place it in specific baskets on their hind legs). The type of bee pollen can be determined, for example, on the basis of its color, ranging from light yellow to black and depending on the type of plant source. According to Campos et al.^3^, monofloral bee pollen is defined as pollen in which the dominant pollen type (taxon) constitutes at least 80% of the total pollen grains present. This classification is based on palynological (microscopic) analysis, which identifies and quantifies the botanical origin of the pollen grains.

Bee pollen is increasingly recognized as a potent source of neuroprotective and antioxidant compounds, notably flavonoids such as quercetin, which have demonstrated significant potential in mitigating oxidative stress and inflammation associated with neurodegenerative diseases. Quercetin, abundant in bee pollen, can cross the blood-brain barrier and has been shown to modulate signaling pathways,- enhancing the brain’s antioxidant defenses and reducing neuronal damage^4^. The antioxidant capacity of bee pollen is due to some of its components, such as phenolics, glutathione, vitamins C and E, etc.^5^; reducing the concentration of reactive oxygen species and remove free radicals. These bioactive compounds are the basis of the treatment of cardiovascular and degenerative diseases (arthritis, Parkinson’s and Alzheimer’s) and diabetes. In addition, phenolics have anticancer, hepatoprotective and detoxifying properties^6^. They stimulate the secretion of tumor necrosis factor alpha, strengthen the immune system and improve liver function. In addition, flavonoids bind heavy metals^7^. Aromatic substances contained in bee pollen (mainly anethole), fatty acids, phytosterols and phenolics (mainly quercetin) have anti-inflammatory effects. These compounds lower the level of prostaglandins, remove cardiovascular and renal edema. In addition, they improve the condition of patients with asthma and strengthen the immune system^8^. Glucose oxidase, flavonoids and phenolic acids present in bee pollen degrade the cytoplasmic membranes of bacteria and fungi. In addition, free omega-3 fatty acids and α-linolenic acid have anti-atherosclerotic and anti-stroke effects. They reduce the absorption of total lipids, triacylglycerols, dietary cholesterol and prevent platelet aggregation, which helps to avoid stroke. Flavonoids, steroids and volatile oil compounds have an immunological regulatory and anti-allergic effect; they inhibit mast cell activity and histamine secretion^7^.

Bee pollen is used as a dietary supplement, additive to food, fodder and cosmetics. It is added, for example, into juices, baked goods (e.g., bread), confections (e.g., cookies) and meat products (e.g., sausages). It is mainly included in the product formula due to its beneficial health effects; however, it has other benefits. For instance, the addition of bee pollen (i) into wines or meads increases ethanol production, (ii) in the production of fermented milk products increases the viability of probiotic bacteria; (iii) into yogurt improves their texture; (iv) into meat products creates and stabilizes emulsions and absorbs water and fat; and (v) into feed partially replaces antibiotics^9^.

Currently, consumers pay more attention to the composition of the products they consume. In addition to sensory properties, the composition and possible health-promoting effects of food became important drivers determining food buying and consumption. This resulted in the intensification of the search for new snack additives with potential beneficial effects on the human body^10^. Studies were carried out on the addition of different functional ingredients into potato snacks: fresh chokeberry^11^, cricket flour^12^, leek and onion^13^, and beetroot pulp^14^. Each of these additives improved the nutritional value of the snacks. For instance, the addition of chokeberry decreased the fat absorption index^11^, while the addition of beetroot or cricket flour increased the content of crude protein, crude fiber and total polyphenol^11,12^. On the other hand, the addition of onion or leek had a significant effect on the texture of the snacks, increasing their hardness^13^.

The aim of the study was to investigate chemical composition, antioxidant activity and color of bee pollens (rapeseed *(Brassica napus* L.*)*, heather (*Calluna vulgaris* (L.) Hull), buckwheat *(Fagopyrum sp.)*, multifloral, phacelia *(Phacelia sp.)*, and multifloral with a predominance of *Papaver sp.*) and the effect of introducing selected bee pollens (rapeseed, multifloral, *Phacelia sp*., and multifloral with a predominance of *Papaver sp.*) in the amount of 1, 3 and 5% into the formulation of fried potato snacks on the content of total phenolics, total flavonoids, antioxidant activity and physicochemical and sensory properties of the obtained snacks. Moreover, an additional aim was to investigate the effect of the snack production process (extrusion and frying) on total polyphenols and flavonoids content and antioxidant activity in comparison to the mixture from which they were produced.

## Materials and methods

### Raw material

**Fig. 1:**
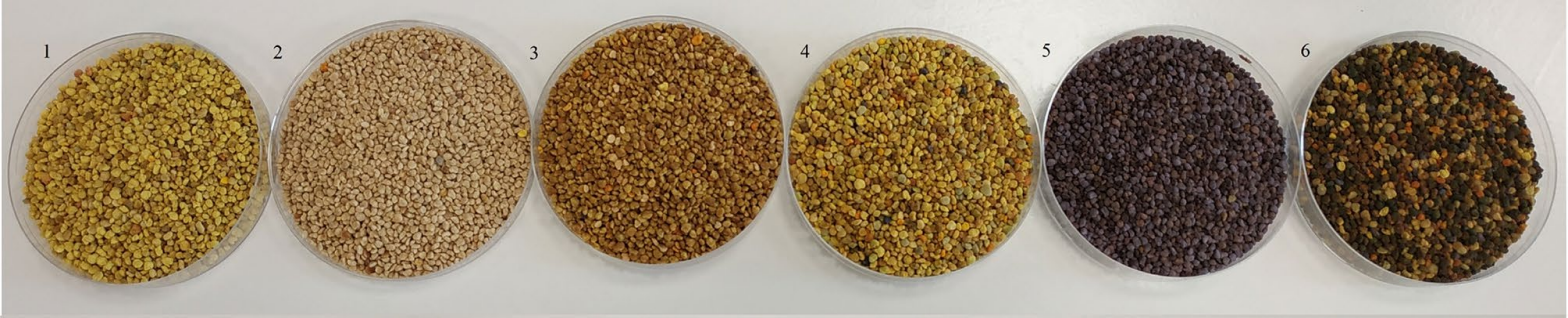
Bee pollens used in experiment: 1 - rapeseed, 2 - Heather, 3 – Buckwheat, 4 – mulfiloral, 5 – *Phacelia* Sp., 6 – with strong dominance of *Papaver* Sp.

The raw material used in the study were six types of bee pollens: (i) rapeseed (RS), (ii) heather (HE), (iii) buckwheat (BW), (iv) multifloral (MF), (v) multifloral with a predominance of*P apaver sp*. bee pollen (MP), and (vi) phacelia *(Phacelia sp.)* (PH). Rapeseed and multifloral with a predominance of *Papaver sp.* bee pollen came from the “Miodolandia” apiary of Mateusz and Mirella Browarczyk. Heather, buckwheat and multifloral came from “Apiary with passion Harwan” from Miłogoszcz. *Phacelia sp*. bee pollen was purchased from the “Maja” beekeeping farm of R. K. Bondaruk in Czarne Wielkie. The type of bee pollen was declared and confirmed by the supplier, who guaranteed its botanical origin and compliance with the specified monofloral type.The bee pollens used in the experiment are shown in Fig. 1.

The four bee pollens with the highest content of total phenolics were used as an additional raw material for the production of potato snacks, at three addition levels (1, 3 and 5%) while; control samples were snacks without bee pollen addition. This extra-ingredient was mixed with the rest of materials for the production of pellets and the snacks obtained therefrom.

### Condition of pellets extrusion and snacks production

The dough for the production of pellets consisted of potato starch (67%), potato grits (26%), corn flour (5%) and salt (2%). Water was added to the dough until reaching a moisture content of about 40%. Bee pollen was added in the amount of 1%, 3% and 5% in relation to the amount of the basic mixture.

**Fig. 2:**
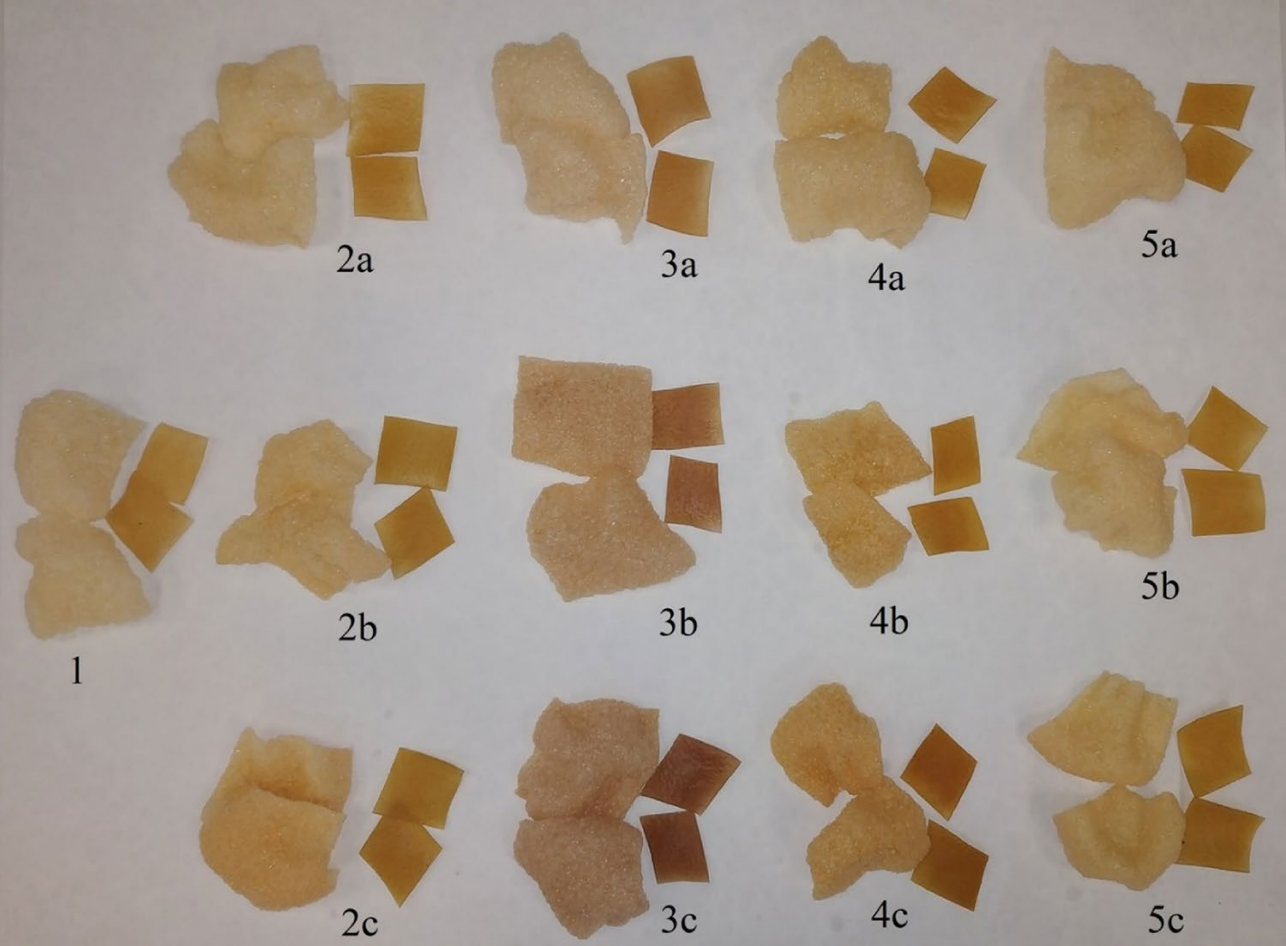
Potato pellets and snacks with the addition of selected bee pollen in various amounts: 1 – controls ample without addition of bee pollen, 2 – rapeseed, 3 – Phacelia Sp., 4 – multifloral with a strong dominance of Papaver Sp. 5 – multifloral; degree of addition: a – 1%, b – 3%, c – 5%.

Prepared dough in a single-screw extruder (Brabender DN 20, Germany) was extruded according to the parameters described by Nemś et al.^15^ and Nemś and Pęksa^16^. Obtained not expanded strands were cut into pellets of ca. 27 × 27 mm and dried at 20 ± 2 °C for 16 h to approximately 10% of moisture content and then sealed in polyethylene bags to equilibrate moisture until frying. Each sample of pellets was fried in a fresh portion of refined rapeseed oil at 185^°^C for 15 s. The product: oil ratio was kept at 1:20 (weight/volume, w/v). After defatting on tissue-paper and cooling, the snacks were packed in metalized bags under air atmosphere^15,16^. Experiment was conducted in two technological repetitions. Obtained snacks and pellets are shown in Fig. 2.

### Analysis

#### Proximate chemical composition analysis

The dry matter, total protein, fat, and ash content of bee pollens were determined according Association of Official Analytical Chemist’ methods (AOAC)^17^. Total soluble and reducing sugars were determined by colorimetric method with the use of 3,5-dinitrosalicylic acid^18^. The content of sucrose was calculated as the difference between total and reducing sugars multiply by 0.95.

#### Preparation of extracts for total phenolics flavonoids and antioxidant activity determination

The samples were extracted in 80% aqueous methanol in a graduated tube. The mixtures were homogenised using a vortex for 5 s, agitated in an ultrasonic bath for 30 min and shaken for 60 min. After 22 h of storage at − 22 °C, the prepared solutions were again shaken for 90 min and centrifuged (at 10,000 rpm for 10 min at 4 °C). The remaining extracts were brought to a known volume with 80% methanol and stored at − 20 °C until further analysis^19^. In bee pollen and snacks, total phenolics, total flavonoids content and antioxidant capacity, as di(phenyl)-(2,4,6-trinitrophenyl)iminoazanium (DPPH•), 2,2′-azino-bis(3ethylbenzthiazoline-6-sulphonic acid) (ABTS•+) and the ferric reducing antioxidant power (FRAP) were also determined.

#### Total phenolic content (TPC)

The total phenolics content was examined in 80% methanol extracts (v/v) using Folin- Ciocalteu colorimetric method as described by^20^. Results (*n* = 6) were expressed as mg gallic acid equivalent (GAE) per 1 g of dry matter (d.m.) of snacks.

#### Total flavonoid content (TFC)

The total flavonoid content was analyzed in 80% methanol extracts (v/v) and was performed using a spectrophotometric method developed by Kim et al.^21^. 0.2 mL of the sample or standard solution, 0.12 mL of 5% NaNO_2_ and 0.12 mL of 10% AlCl_3_ were added to 2.8 mL of water; then, samples were mixed at 25 °C for 5 min. Then 0.8 mL of 1 M NaOH was added. The absorbance of the sample was measured at 510 nm against the blank. The content of flavonoids was measured on the basis of a standard curve, using catechin as a standard. Results (*n* = 6) were expressed as mg catechin (CE) per 1 g of dry matter (d.m.) of snacks.

#### Antioxidant activity

The antioxidant activity was analyzed in 80% methanolic extract (v/v) using the Trolox equivalent antioxidant capacity (TEAC) using the ABTS^•+^^22^, DPPH^•^^23^ and FRAP^24^ methods. Results (*n* = 6) were expressed as µmol TROLOX per 1 g of dry matter (d.m.) of snacks.

#### Determination of phenolic profile by UPLC-PDA-MS/MS

Samples (1 g) were extracted with 5 mL of a mixture containing UPLC-grade methanol (30%) and acetic acid (1% of reagent). The extraction was performed twice by incubation for 20 min under sonication (Sonic 6D, Polsonic, Warsaw, Poland) and with occasional shaking. Next, the slurry was centrifuged at 19,000 g for 10 min, and the supernatant was filtered through a hydrophilic PTFE 0.20 μm membrane (Millex Samplicity Filter, Merck, Darmstadt, Germany) and used for analysis. Determination of phenolic compounds was carried out using the UPLC apparatus equipped with a binary pump, column and sample manager, photodiode array detector (PDA), and tandem quadrupole mass spectrometer (TQD) with electrospray ionization (ESI) source working in negative mode (Waters, Milford, MA, USA) according to the method of Żurek et al.^25^. Separation was performed using the UPLC BEH C18 column (1.7 μm, 100 mm × 2.1 mm, Waters) at 50 °C, at an isocratic flow rate of 0.35 mL/min. The injection volume of the samples was 5 µL. The mobile phase consisted of water (solvent A) and 40% acetonitrile in water, v/v (solvent B). The following TQD parameters were used: capillary voltage of 3500 V; con voltage of 30 V; con gas flow 100 L/h; source temperature 120 °C; desolvation temperature 350 °C; and desolvation gas flow rate of 800 L/h. Polyphenolic identification and quantitative analyses were performed on the basis of the mass-to-charge ratio, retention time, specific PDA spectra, fragment ions, and comparison of data obtained with literature findings and commercial standards. The results were expressed as mg per 1 g dry matter (d.m.)^25^.

#### Color of snacks

The snacks color was expressed using the CIEL*a*b* space, lightness (L*), a* (green-red coordinate) and b* (blue-yellow coordinate), as well as the hue angle (*h*°) and chroma (*C*). These color parameters were obtained using a Konica Minolta CM-5 spectrophotometer (Osaka, Japan). Analysis was conducted in five repetitions on milled samples. ΔE was determined using a “CIE 2000 calculator” (http://colormine.org/delta-e-calculator/cie2000)^26^;.

#### Texture

The texture of the snacks was evaluated using an Instron type 5544 texture-measuring device with a Bluehill universal software. The minimal force (N) necessary to break up a snack was recorded using a shear blade working at a displacement rate of 250 mm/min. Data reported is the mean of 20 measurements^26^.

#### Bulk weight

In order to determine the bulk weight of the snacks, samples were placed into a 1 L measuring cylinder. The whole was shaken to get rid of empty spaces, and if necessary, more snacks were added until completing the volume of 1 L. The sample measured with a cylinder was weighed with an accuracy of 0.01 g. The determination was conducted in five repetitions and results are expressed g per 1 L^27^.

#### Expansion index

To determine the index of expansion of the snacks, the thickness of the pellets was measured in three different places. Then, the pellets were fried and the thickness of the fried snacks was measured in the same places. The measurement was made using a digimatic caliper (Mitutoyo, Japan). The assay was performed in ten replicates^16^.

#### Sensory evaluation

The evaluation of the sensory properties of the snacks was carried out according to the methodology PN-A-88036^28^. A trained 8-member sensory panel (25–35 years of age; 40% males and 60% females), rated the sensory quality of snacks on the following attributes: color, texture, structure, odor and flavor.

The samples of snacks were placed in pots marked with 3-digit codes. A 7-point grading scale was used and the scorecard is shown in Appendix 1, where 1: low quality, far from the established requirements; and, 7: high quality, close to the established requirements. The evaluated attributes were:

- Color evaluation included the intensity and uniformity of the snacks color.
- Appearance evaluation included the uniform expansion and surface corrugation of the snacks.
- Texture evaluation included the crispiness and hardness of the snacks.
- Odor and flavor evaluation included the occurrence of the typical notes of this specific type of snacks and the occurrence of off-flavors (e.g., rancid and burnt notes) with the sample out- or in-side the mouth, respectively.

Research was approved by the Rector’s committee for research ethics (Wrocław University of Environmental and Life Sciences) under the reference N0N00000.0011.5.2024.

### Statistical analysis

Statistical analysis of all data was performed using a one-way analysis of variance (ANOVA), with factor being the bee pollen type. The Duncan’s multiple range test was used to determine the differences among samples with a probability level of α = 0.05. Moreover, a dendrogram analysis (using Euclidean distance by Ward method) and principal components analysis (PCA) were determined. Statistical analyses and standard deviations were determined using Statistica v. 13.0 software (StatSoftinc., Tulsa, OK, USA).

## Results and discussion

### Chemical composition of bee pollens used in experiment

The analyzed bee pollens differed in terms of basic chemical composition (Table 1). Bee pollens contained from 6.96 to 13.37% of water, which is more than suggested 6%^3,29^. ISO standards allows the moisture of dried bee pollen to be at a level 2–8%^30^. Too high moisture may cause the development of undesirable bacteria and mold^31^. The average content of protein (13.96–27.84%), fat (5.80–12.76%), ash (2.39–4.42%) and total sugars (33.42–49.39%) in the tested bee pollens were within the ranges reported previously by other authors^2,29,32^. The analyzed bee pollen contained from 29.58% (RS) to 33.33% (HE) of reducing sugars and from 33.42% (RS) to 49.39% (HE) total sugars. The total sugars content is the most important quality parameter and should not be less than 40%^8^. Compared to the others bee pollens (from 3.65 to 7.72%), heather bee pollen contained much more sucrose, as much as 15.26% (Table 1). Kędzia and Hołderna-Kędzia^31^ found that the average content of non-reducing sugars like sucrose in bee pollen was 3.4%. The composition of bee pollen mainly depends on the type of plant from which it was collected, but it also influenced by climate, soil type, harvesting season, geographical origin as well as storage methods^8^.

**Table 1:** Chemical composition and antioxidant activity of the bee pollen samples used in experiment.

Parameter	Bee pollen type						
	Rapeseed, RS	Heather, HE	Buckwheat, BW	Multiflorous, MF	Phacelia, PH	Multiflorous with papaver, MP	LSD*
Dry matter [%]	90.85 $\:\pm\:\:$0.02 ^d^	93.04 $\:\pm\:\:$0.03 ^a^	91.13 $\:\pm\:\:$0.03 ^c^	90.01 $\:\pm\:\:$0.07 ^e^	86.63 $\:\pm\:\:$0.05 ^f^	91.65 $\:\pm\:\:$0.04 ^b^	0.10
Protein [%]	25.97 $\:\pm\:\:$0.49 ^e^	17.73 $\:\pm\:\:$0.29 ^c^	13.96 $\:\pm\:\:$0.67 ^d^	23.48 $\:\pm\:\:$0.18 ^b^	27.84 $\:\pm\:\:$0.42 ^a^	26.71 $\:\pm\:\:$0.07 ^e^	0.80
Fat [%]	12.76 $\:\pm\:\:$0.12 ^a^	5.80 $\:\pm\:\:$0.00 ^f^	8.07 $\:\pm\:\:$0.08 ^c^	9.75 $\:\pm\:\:$0.03 ^b^	6.03 $\:\pm\:\:$0.04 ^e^	7.70 $\:\pm\:\:$0.05 ^d^	0.16
Ash [%]	2.74 $\:\pm\:\:$0.01 ^e^	2.39 $\:\pm\:\:$0.03 ^d^	4.42 $\:\pm\:\:$0.00 ^a^	2.70 $\:\pm\:\:$0.02 ^e^	2.82 $\:\pm\:\:$0.02 ^b^	2.57 $\:\pm\:\:$0.01 ^c^	0.04
Total sugars [%]	33.42 $\:\pm\:\:$0.24 ^f^	49.39 $\:\pm\:\:$0.26 ^a^	39.17 $\:\pm\:\:$0.27 ^c^	35.84 $\:\pm\:\:$0.20 ^e^	37.10 $\:\pm\:\:$0.23 ^d^	39.98 $\:\pm\:\:$0.15 ^b^	0.34
Reducing sugars [%]	29.58 $\:\pm\:\:$0.09 ^f^	33.33 $\:\pm\:\:$0.09 ^a^	31.25 $\:\pm\:\:$0.09 ^c^	30.21 $\:\pm\:\:$0.10 ^e^	30.54 $\:\pm\:\:$0.10 ^d^	31.86 $\:\pm\:\:$0.06 ^b^	0.13
Sucrose [%]	3.65 $\:\pm\:\:$0.31 ^d^	15.26 $\:\pm\:\:$0.26 ^a^	7.53 $\:\pm\:\:$0.30 ^e^	5.35 $\:\pm\:\:$0.20 ^c^	6.24 $\:\pm\:\:$0.22 ^b^	7.72 $\:\pm\:\:$0.13 ^e^	0.37
Total phenolics [mg GAE/g d. m.]	20.51 $\:\pm\:\:$0.26 ^b^	7.48 $\:\pm\:\:$0.10 ^e^	3.03 $\:\pm\:\:$0.03 ^f^	13.77 $\:\pm\:\:$0.27 ^c^	29.65 $\:\pm\:\:$0.28 ^a^	13.16 $\:\pm\:\:$0.09 ^d^	0.23
Total flavonoids [mg CE/g d.m.]	8.82 $\:\pm\:\:$0.31 ^a^	1.87 $\:\pm\:\:$0.05 ^e^	1.48 $\:\pm\:\:$0.15 ^f^	8.18 $\:\pm\:\:$0.28 ^b^	7.26 $\:\pm\:\:$0.33 ^c^	5.80 $\:\pm\:\:$0.28 ^d^	0.30
ABTS^+•^ [µmol Trolox/g d.m]	217.2 $\:\pm\:\:$9.15 ^d^	106.3 $\:\pm\:\:$1.6 ^e^	53.53 $\:\pm\:\:$1.46 ^f^	234.5 $\:\pm\:\:$4.4 ^c^	349.9 $\:\pm\:\:$10.1 ^a^	257.3 $\:\pm\:\:$6.3 ^b^	7.59
DPPH^•^ [µmol Trolox/g d.m.]	66.16 $\:\pm\:\:$2.40 ^a^	6.44 $\:\pm\:\:$0.30 ^d^	6.84 $\:\pm\:\:$0.48 ^d^	59.68 $\:\pm\:\:$2,09 ^b^	19.07 $\:\pm\:\:$0.97 ^c^	17.49 $\:\pm\:\:$0.62 ^c^	1.65
FRAP [µmol Trolox/g d.m.]	53.39 $\:\pm\:\:$2.27 ^c^	3.96 $\:\pm\:\:$0.43 ^d^	4.75 $\:\pm\:\:$0.14 ^d^	51.40 $\:\pm\:\:$3.62 ^c^	42.65 $\:\pm\:\:$3.33 ^a^	38.00 $\:\pm\:\:$1.17 ^b^	2.67

Values are mean ± SD of three determinations (dry matter, protein, fat, ash, total sugars, succrose), six determinations (total polyphenols, total flavonoids, ABTS, DPPH, FRAP).

Values followed by the same letter, within the same row, were not significantly different (*p* > 0.05), according to Duncan’s multiple range test.

Bee pollens used in the experiment contained from 3.03 mg GAE/g d.m.(BW) to 20.51 mg GAE/g d.m. (RS) of total phenolics and from 1.48 mg CE/g d.m. (BW) to 8.82 mg CE/g d.m (RS) of total flavonoids. Kędzia and Hołderna-Kędzia^31^ found that the average content of total phenolics in bee pollen is 1.29%, and total flavonoids 0.67%. Bee pollens studied by Leja et al.^33^ contained from 1.3 to 8.2% of total phenolics and from 0.2 to 1.4% of total flavonoids. The percentage composition of bee pollen depends to the greatest extent on the type of plant from which it was collected^32^.

The antioxidant capacity measured through ABTS^•+^ of bee pollen was ranged from 53.53 (BW) to 349.87 µmol Trolox/g d.m. (PH); DPPH^•^ from 6.44 (HE) to 66.16 µmol Trolox/g d.m. (RS); and FRAP from 3.96 (HE) to 53.39 µmol Trolox/g d.m. (RS) (Table 1). The higher antioxidant activity observed in the ABTS assay compared to the DPPH assay suggests that hydrophilic antioxidant compounds are more abundant in the analyzed bee pollen samples. This aligns with the known properties of the ABTS radical cation, which is more sensitive to both hydrophilic and lipophilic antioxidants, but particularly effective in detecting water-soluble compounds, whereas DPPH radical primarily interacts with lipophilic substances^34^. Rocchetti et al.^35^ studied bee pollen and showed that their antioxidant activity ABTS^•+^ ranged from 48.8 to 224.6 µmol Trolox/g d.m., and DPPH^•^ from 11.9 to 134.7 µmol Trolox/g d.m. Studies on the antioxidant activity of FRAP showed that it can range from 11.77 to 105.06 µmol Trolox/g d.m^36^. It was noted that the antioxidant activity of the tested bee pollen depended on the botanical origin of the plant from which it was collected, similar conclusions were drawn by Rocchetti et al.^35^. As shown in multiple studies, there are significant differences in antioxidant activity, chemical component concentration, and types among bee pollen grains from different plant species and geographical locations. The type of plant from which bee pollen originates, the growing conditions of the plants, such as soil or climate, and the time of harvest, affect both the content and the characteristics of the bee pollen^2^.

In general, it is normally considered that the antioxidant activity of bee pollen depends on its phenolics content. The calculated correlation coefficients between the TPC and individual antioxidant activities were: ABTS^•+^ 0.86, DPPH^•^ 0.64; FRAP 0.88, and between the TFC and the individual antioxidant activities were: ABTS^•+^ 0.86; DPPH^•^ 0.84; FRAP 0.99. As can be seen, the higher the content of both TPC and TFC, the higher the antioxidant activity of the bee pollens, which agrees with findings by other authors^37,38^.

On the other hand, *Phacelia sp.* bee pollen was characterized by the highest ABTS^•+^ antioxidant activity, despite the fact that it did not contain the largest amount of phenolics (Table 1). This may suggest that the antioxidant capacity of ABTS^•+^ was influenced not only by the amount of phenolics, but also by other factors. Peptides with free histidine and tyrosine amino groups and some free amino acids, e.g., methionine and arginine, also have antioxidant properties^39^. Researchers suspect that the antioxidant activity also depends on the composition of the phenolic fraction, because the compounds contained in this fraction show different antioxidant activity^40^. Other compounds found in bee pollen—such as carotenoids, glutathione, phytoalexins, and vitamins C and E—also play an important role in enhancing its antioxidant potential. These molecules work alongside polyphenols to protect cells from oxidative stress and support the body’s overall defense against free radicals^2^. Czerwonka et al.^37^ suspect that the color of bee pollen can be linked with its antioxidant activity in the way that darker colors are related to higher antioxidant activity. It is based on research conducted on honeys, in which it turned out that dark red honeys (containing large amounts of anthocyanins) were characterized by higher antioxidant activity than the rest of the honeys tested^41^. Among the tested bee pollens, the darkest color (Table 2) and the highest antioxidant activity ABTS^•+^ were found in *Phacelia sp*. bee pollen, followed by multifloral-papaver bee pollen. Leja et al.^33^ reported that the *Phacelia sp.* bee pollen contained the highest anthocyanins content among all the bee pollens used in their experiment (0.33 mg per 1 g of bee pollen) and was characterized by one of the highest antioxidant activity.

**Table 2:** Color coordinates of the bee pollen samples used in experiment.

Parameter	Bee pollen type						LSD*
	Rapeseed, RS	Heather, HE	Buckwheat, BW	Multiflorous, MF	Phacelia, PH	Multiflorous with papaver, MP	
L*	73.88$\:\pm\:\:$0.06 ^b^	74.57$\:\pm\:\:$0.16 ^a^	66.73$\:\pm\:\:$0.08 ^d^	69.59$\:\pm\:\:$0.14 ^c^	53.21$\:\pm\:\:$0.06 ^f^	55.85$\:\pm\:\:$0.19 ^e^	0.16
a*	1.73$\:\pm\:\:$0.03 ^f^	1.96$\:\pm\:\:$0.03 ^e^	3.69$\:\pm\:\:$0.04 ^b^	3.97$\:\pm\:\:$0.06 ^a^	2.69$\:\pm\:\:$0.04 ^c^	2.03$\:\pm\:\:$0.02 ^d^	0.05
b*	39.00$\:\pm\:\:$0.11 ^a^	22.11$\:\pm\:\:$0.06 ^d^	29.57$\:\pm\:\:$0.13 ^c^	35.00$\:\pm\:\:$0.16 ^b^	3.45$\:\pm\:\:$0.06 ^f^	17.04$\:\pm\:\:$0.16 ^e^	0.16
*C*	39.03$\:\pm\:\:$0.11 ^a^	22.20$\:\pm\:\:$0.06 ^d^	29.83$\:\pm\:\:$0.12 ^c^	35.10$\:\pm\:\:$0.09 ^b^	4.38$\:\pm\:\:$0.06 ^f^	17.16$\:\pm\:\:$0.16 ^e^	0.14
*h*°	87.46$\:\pm\:\:$0.04 ^a^	84.93$\:\pm\:\:$0.09 ^b^	82.90$\:\pm\:\:$0.15 ^e^	83.53$\:\pm\:\:$0.13 ^c^	51.98$\:\pm\:\:$0.51 ^d^	83.19$\:\pm\:\:$0.08 ^e^	0.30

Values are mean ± SD of five determinations.

Values followed by the same letter, within the same row, were not significantly different (*p* > 0.05), according to Duncan’s multiple range test.

During chromatographic analysis of the bee pollens by UPLC-PDA-MS/MS, the total number of compounds found in each of the bee pollen types were 32 in multifloral with strong predominance of *Papaver sp*., 25 in multifloral, 23 in *Phacelia sp*., 15 in rapeseed, 14 in heather, as well as 9 in buckwheat (Table 3). The compounds detected in analyzed bee pollen were mainly derivatives of quercetin, kaempferol, isorhamnetin and spermidine. Komosińska-Vassev et al.^1^, Serra Bonvehi, Soliva Torrento, and Centelles Lorente^42^, also detected in bee pollens derivatives of kaempferol, quercetin as well as isorhamnetin.

**Table 3:** Individual phenolic compounds identified by UPLC-PDA-MS/MS of the beepollen samples used in experiment.

Compound	Rt (min)	λ_max_ (nm)	[M-H] m/z		Bee pollen type					
					Rapeseed, RS	Heather, HE	Buckwheat, BW	Multiflorous, MF	Phacelia, PH	Multiflorous with papaver, MP
			MS	MS/MS						
					Content (mg/g)					
Caffeoyl-tartaric acid	1.76	293sh, 324	311	179			0.066			
Caffeoyl-glucaric acid	2.5	299sh, 327	371	209			0.169			
(+)catechin	2.96	273	289	-		0.005				
Pelargonidin 3-*O*-rutinoside	2.97	278, 515	579	271						0.07
Chlorogenic acid	3.05	299sh, 327	353	179		0.05				
Coumaric acid glucoside	3.27	309	325	163			0.05			
Kaempferol 3-*O*-sophoroside-7-*O*-glucoside	3.52	264, 347	771	609, 285				0.02		
Isorhamnetin 3-*O*-sophoroside-7-*O*-glucoside	3.77	254, 354	801	639, 315				0.05		
Quercetin 3-*O*-glucoside-7-*O*-glucoside	3.91	255, 355	625	463, 301				0.05		
Quercetin 3-*O*-sophoroside	4.06	255, 355	625	301	1.38	0.02		0.59		0.25
Kaempferol 3-*O*-glucoside-7-*O*-glucoside	4.16	264, 338	609	447, 285	0.72			0.2		0.02
Quercetin 3-*O*-rutinoside-7-*O*-rhamnoside	4.2	255, 354	755	609, 301					0.25	
Kaempferol 3-*O*-sophoroside-7-*O*-glucoside	4.31	264, 347	771	609, 285			0.035			
Kaempferol 3-*O*-sophoroside-7-*O*-glucuronide	4.31	267, 339	785	609, 285				0.07		
*p*-coumaric acid	4.33	309	163	119					0.16	
Quercetin 3-*O*-glucoside-pentoside	4.37	255, 353	595	301						0.74
Quercetin 3-*O*-sophoroside-7-*O*-glucuronide	4.38	255, 350	801	625, 301			0.04			
Isorhamnetin -*O*-sophoroside	4.47	269, 328	639	315		0.09		0.47		0.2
Kaempferol 3-*O*-sophoroside	4.52	264, 347	609	285	1.82	0.03		0.65	0.12	0.92
Kaempferol 3-*O*-rhamnoside	4.53	264, 347	431	285		0.04				
Kaempferol 3-*O*-rutinoside-7-*O*-rhamnoside	4.61	264, 347	739	593, 285					0.75	0.01
Quercetin 3-*O*-rutinoside-7-*O*-glucoside	4.72	255, 353	771	609, 301						0.08
Isorhamnetin 3-*O*-rutinoside-7-*O*-rhamnoside	4.73	253, 354	769	623, 315					2.67	
Kaempferol 3-*O*-sophoroside-7-*O*-glucuronide	4.77	264, 347	785	609, 285			0.04			
Kaempferol 3-*O*-sophoroside-7-*O*-rhamnoside	4.86	264, 348	755	609, 285	0.28	0.71	0.02	0.33		
Kaempferol 3-*O*-glucoside-pentoside	4.9	264, 348	579	285						0.99
Kaempferol 3-*O*-rutinoside	4.95	264, 347	593	285	0.18	0.03		0.33	0.94	0.22
Quercetin 3-*O*-rhamnoside-pentoside-7-*O*-rhamnoside	5.04	255, 352	725	579, 301						0.03
Isorhamnetin 3-*O*-rutinoside	5.11	253, 354	623	315	0.18			0.18	0.82	0.01
Quercetin 3-*O*-rutinoside	5.17	255, 355	609	301	0.08					
Kaempferol 3-*O*-rhamnoside	5.2	264, 346	431	285						0.01
Kaempferol 7-*O*-rhamnoside	5.35	264, 347	431	285		0.01				
Kaempferol 3-*O*-glucoside	5.49	264, 347	447	285	0.25	0.16		0.17	0.26	0.01
Isorhamnetin 3-*O*-(6’-acetyl)-sophoroside	5.56	255, 317	665	623, 315	0.03			0.03		
Isorhamnetin 3-*O*-glucoside	5.68	253, 352	477	315				0.02	0.24	0.04
Kaempferol 3-*O*-rhamnoside-7-*O*-pentoside	5.85	269, 327	563	431, 285	1.35			0.77		0.07
Isorhamnetin 3-*O*-(6”acetyl)-glucoside	5.94	255, 309	519	477, 315	0.33			0.2		0.01
3-*O*-caffeoyl-5-*O*-coumaryloquinic acid	6.03	291sh, 309	499	353, 179					0.67	0,10
Quercetin 3-*O*-rhamnoside-7-*O*-rhamnoside	6.1	255, 352	593	447, 301			0.03	0.44		0.26
*N,N*-di-coumaroyl putrescine isomer I	6.2	291sh, 307	379	146, 87					0.27	0.2
Kaempferol 4’-*O*-rhamnoside	6.25	264, 347	431	285		0.03				
*N,N*-di-coumaroyl putrescine isomer II	6.46	291sh, 309	379	146, 87					0.64	0.12
*N,N*-di-coumaroyl putrescine isomer III	6.66	291sh, 309	379	146, 87					2.67	0.7
*N,N,N*-tris-caffeoyl spermidine	6.53	295sh, 319	630	468, 145				0.74		
*N,N*-dicaffeoyl-*N*-coumaroyl spermidine isomer I	6.91	293sh, 317	614	452, 290, 144					0.21	2.09
*N,N*-dicaffeoyl-*N*-coumaroyl spermidine isomer II	7.02	292sh, 309	614	452, 144					0.41	0.09
*N,N*-dicaffeoyl-*N*-feruloyl spermidine	7.16	299sh, 319	644	468, 145	2.49			1.37		
*N*-caffeoyl-*N,N*-di-coumaroyl spermidine isomer I	7.45	296	598	436, 290				0.45	0.22	0.05
*N*-caffeoyl-*N,N*-di-coumaroyl spermidine isomer II	7.53	296	598	436, 290				0.31	0.86	0.73
*N*-caffeoyl-*N,N*-di-coumaroyl spermidine isomer III	7.45	296	598	436, 290					1.78	0.14
*N*-caffeoyl-*N,N*-di-coumaroyl spermidine isomer IV	7.53	296	598	436, 290	0.34				3.01	0.28
Kaempferol 3-*O*-(6”acetyl)-glucoside-rhamnoside	7.6	264, 316	635	593, 285		1.3	0.12			
*N,N,N*-tris-coumaroyl spermidine isomer I	7.68	271	582	436, 144	0.45			0.43	0.59	0.3
*N,N,N*-tris-coumaroyl spermidine isomer II	7.8	271	582	436, 144				0.48	1.35	0.22
Isorhamnetin 3-*O*-(6”coumaroyl)-glucoside	7.84	253, 328	623	477, 315		0.02				
*N,N,N*-tris-coumaroyl spermidine isomer III	7.86	271	582	436, 144				1.2	2.95	1.07
*N*-caffeoyl-*N*-feruloyl-*N*-coumaroyl spermidine	7.98	295	628	452, 482	1.16			2.29	5.58	1.22
Kaempferol 3-*O*-coumaroyl-rhamnoside	8.08	264, 312	593	431, 285		0,06				
Total					11.01	2.57	0.58	11.87	27.39	11.22

The total concentration of phenolics identified by UPLC-PDA-MS/MS ranged from 0.58 mg/g d.m. (BW) to 27.39 mg/g d.m. (PH) (Table 3). The sum of thes compounds could be ranked as follows: BW < HE < RS ≅ MP ≅ MF < PH. The predominant compound in multifloral-papaver, multifloral and *Phacelia sp.* bee pollens was *N*-caffeoyl-*N*-feruoyl-*N*-coumaroyl spermidine (1.22, 2.29 and 5.58 mg/g d.m., respectively). Moreover in rapeseed bee pollen the most abundant compound was *N, N*-dicaffeoyl-*N*-feruoyl spermidine (2.49 mg/g d.m.), in heather bee pollen was kaempferol 3-*O*-sophoroside-7-*O*-rhamnoside (0.71 mg/g d.m.), and in buckwheat bee pollen was caffeoyl-glucaric acid (0.169 mg/g d.m.). In the studies conducted by Qiao et al.^43^, it was proven that the dominant phenylamides in rapeseed pollen were: *N*^1^, *N*^10^-di-*p*-coumaroyl spermidine (8.33 mg/g), *N*^1^,*N*^5^,*N*^10^-tri-*p*-coumaryl spermine (7.78 mg/g), in corn poppy wheat pollen *N*^1^,*N*^14^-di-*p*-coumaryl-*N*^5^-*N*^10^-di diacetyl spermine (5.53 mg/g), *N*^1^,*N*^14^-di-p-coumaryl-*N*^5^- hydroxyavenalumoyl spermine (3.17 mg/g), while in buckwheat pollen *N*^1^-hydroxyferuloyl-*N*^5^-p-coumaroyl-*N*^14^-feruoyl spermine (1.11 mg/g), tri-*p*-coumaroyl spermidine (0.85 mg/g) and *N*^1^, *N*^10^-di-*p*-coumaroyl-*N*14-hydroxyferuoy spermidine (0.83) were dominant. Moreover, in rapeseed bee pollen the predominant flavonoids were quercetin-3-*O*-sophoroside (7.78 mg/g) and kaempferol-3-*O*-sophoroside (4.19 mg/g), in corn poppy bee pollen kaempferol-3-*O*-sophoroside (10.13 mg/g) and kaempferol-3-*O*-rutinoside (4.02 mg/g), and in buckwheat bee pollen quercetin-3-*O*-arabinglucoside (0.57 mg/g). Recent studies have consistently demonstrated that bee pollen is a rich source of phenylamides. For instance, 18 phenylamides were identified in bee pollen from *Quercus mongolica*^44^, 13 in rapeseed bee pollen (*Brassica napus*) as reported by Qiao, Feng et al.^43^. Additionally, pollen samples collected from Colombia, Italy, and Spain contained 18 phenolamides^45^, while analysis of 20 different types of Chinese bee pollen revealed the presence of 64 phenylamides^43^. Based on current knowledge, a total of 70 distinct phenylamides have been identified in bee pollen so far, representing more than one-third of all phytochemicals detected in this natural product^46^.

Analyzed bee pollens varied in terms of color (Table 2); their color depends on the pigments they contain, for example chlorophylls, carotenoids, flavonoids, and anthocyanins^31^. The color of *Phacelia sp.* bee pollen was very dark, almost black (L*=53,21; *C* = 4.38; and *h*°=51.98) and perhaps its color is linked to its anthocyanins profile, which can be characterized by dark colors^33^. On the other hand, the color of the heather sample was the lightest one and it was beige (L*=74.57; *C* = 22.20; *h*°=84.93). Bee pollens of rapeseed, buckwheat and multifloral samples were of orange colors, probably linked to their carotenoids profiles^32^.

### The effect of pellet snack processing on total phenolics, flavonoids and antioxidant activity of ready-to-eat products

The four bee pollens with the highest content of total phenolics (RS, MF, MP, PH) were used as an additional raw material for the production of potato snacks. The addition of bee pollen significantly increased the TPC and TFC in the mixtures for the preparation of pellets (Table 4) as compared to the control sample. As expected, the richest bee pollens in phenolics led to obtain mixtures with the highest phenolic content. With the increasing degree of bee pollen addition to the mixtures, the TPC and TFC increased. The addition of bee pollen also increased the antioxidant capacity (ABTS^•+^, DPPH^•^ and FRAP) of the pellet mixtures (Table 5). The higher the addition of bee pollen, the higher the antioxidant activity of the mixtures, regardless of the test performed. On average, mixtures containing rapeseed bee pollen were characterized by the highest antioxidant activity (Table 4).

**Table 4:** Total polyphenols and flavonoids contents and antioxidant activity of dough and snacks with selected bee pollen.

Bee pollen type	Level of addition [%]	Total phenolics [mg GAE/g dm]		Total flavonoids [mg CE/g dm]		Antioxidant activity [µmol TROLOX/g dm]					
						ABTS^·+^		DPPH^·^		FRAP	
		Dough	Snacks	Dough	Snacks	Dough	Snacks	Dough	Snacks	Dough	Snacks
Control	0	0.21 $\:\pm\:$0.01 ^j^	0.20 $\:\pm\:$ 0.00 ^k^	0.13 $\:\pm\:$0.01 ^h^	0.04 $\:\pm\:$ 0.01 ^h^	0.49 $\:\pm\:$0.08 ^h^	0.67 $\:\pm\:$ 0.07 ^h^	1.46 $\:\pm\:$0.03 ^k^	1.33 $\:\pm\:$ 0.02 ^i^	0.30 $\:\pm\:$0.03 ^i^	0.26 $\:\pm\:$ 0.02 ^i^
RS	1	0.51 $\:\pm\:$0.01 ^g^	0.26 $\:\pm\:$0.02 ^j^	0.34 $\:\pm\:$0.02 ^f^	0.08 $\:\pm\:$0.01 ^fg^	1.80 $\:\pm\:$0.12 ^f^	1.08 $\:\pm\:$0.15 ^g^	1.97 $\:\pm\:$0.02 ^f^	1.45 $\:\pm\:$0.01 ^g^	1.46 $\:\pm\:$0.04 ^f^	0.47 $\:\pm\:$0.02 ^h^
	3	0.91 $\:\pm\:$0.03 ^d^	0.38 $\:\pm\:$0.02 ^e^	0.58 $\:\pm\:$0.03 ^c^	0.13 $\:\pm\:$0.01 ^d^	5.13 $\:\pm\:$0.06 ^b^	1.41 $\:\pm\:$0.21 ^de^	2.53 $\:\pm\:$0.03 ^c^	1.63 $\:\pm\:$0.02 ^c^	2.15 $\:\pm\:$0.05 ^d^	0.91 $\:\pm\:$0.07 ^d^
	5	1.35 $\:\pm\:$0.02 ^a^	0.47 $\:\pm\:$0.01 ^b^	0.72 $\:\pm\:$0.02 ^a^	0.19 $\:\pm\:$0.01 ^a^	7.14 $\:\pm\:$0.19 ^a^	2.21 $\:\pm\:$0.15 ^b^	2.86 $\:\pm\:$0.01 ^a^	1.82 $\:\pm\:$0.03 ^a^	2.63 $\:\pm\:$0.02 ^c^	1.28 $\:\pm\:$0.04 ^b^
MF	1	0.40 $\:\pm\:$0.01 ^i^	0.31 $\:\pm\:$0.01 ^h^	0.26 $\:\pm\:$0.01 ^g^	0.08 $\:\pm\:$0.01 ^fg^	1.41 $\:\pm\:$0.20 ^g^	1.18 $\:\pm\:$0.17 ^fg^	1.73 $\:\pm\:$0.03 ^h^	1.34 $\:\pm\:$0.01 ^i^	0.96 $\:\pm\:$0.04 ^g^	0.62 $\:\pm\:$0.05 ^g^
	3	0.87 $\:\pm\:$0.02 ^e^	0.35 $\:\pm\:$0.00 ^f^	0.55 $\:\pm\:$0.01 ^d^	0.11 $\:\pm\:$0.01 ^e^	3.95 $\:\pm\:$0.27 ^d^	1.58 $\:\pm\:$0.07 ^d^	2.32 $\:\pm\:$0.03 ^d^	1.55 $\:\pm\:$0.03 ^d^	1.99 $\:\pm\:$0.02 ^e^	0.84 $\:\pm\:$0.03 ^e^
	5	1.17 $\:\pm\:$0.03 ^c^	0.52 $\:\pm\:$0.02 ^a^	0.67 $\:\pm\:$0.01 ^b^	0.16 $\:\pm\:$0.01 ^c^	4.61 $\:\pm\:$0.12 ^c^	2.46 $\:\pm\:$0.17 ^a^	2.74 $\:\pm\:$0.05 ^b^	1.71 $\:\pm\:$0.01 ^b^	3.07 $\:\pm\:$0.09 ^b^	1.45 $\:\pm\:$0.04 ^a^
MP	1	0.42 $\:\pm\:$0.02 ^i^	0.28 $\:\pm\:$0.01 ^i^	0.25 $\:\pm\:$0.02 ^g^	0.07 $\:\pm\:$0.01 ^g^	1.34 $\:\pm\:$0.19 ^g^	1.11 $\:\pm\:$0.17 ^g^	1.63 $\:\pm\:$0.04 ^j^	1.40 $\:\pm\:$0.02 ^h^	0.80 $\:\pm\:$0.08 ^h^	0.62 $\:\pm\:$0.08 ^g^
	3	0.73 $\:\pm\:$0.02 ^f^	0.32 $\:\pm\:$0.00 ^g^	0.44 $\:\pm\:$0.02 ^e^	0.09 $\:\pm\:$0.01 ^f^	2.41 $\:\pm\:$0.10 ^e^	1.39 $\:\pm\:$0.17 ^e^	1.88 $\:\pm\:$0.02 ^g^	1.43 $\:\pm\:$0.01 ^g^	1.93 $\:\pm\:$0.18 ^e^	0.69 $\:\pm\:$0.05 ^f^
	5	0.92 $\:\pm\:$0.01 ^d^	0.41 $\:\pm\:$0.01 ^c^	0.56 $\:\pm\:$0.02 ^d^	0.12 $\:\pm\:$0.01 ^e^	3.78 $\:\pm\:$0.21 ^d^	1.91 $\:\pm\:$0.18 ^c^	2.09 $\:\pm\:$0.02 ^e^	1.48 $\:\pm\:$0.02 ^f^	2.70 $\:\pm\:$0.06 ^c^	0.96 $\:\pm\:$0.04 ^d^
PH	1	0.47 $\:\pm\:$0.03 ^h^	0.27 $\:\pm\:$0.01 ^ij^	0.27 $\:\pm\:$0.01 ^g^	0.08 $\:\pm\:$0.01 ^fg^	1.27 $\:\pm\:$0.18 ^g^	1.29 $\:\pm\:$0.19 ^ef^	1.67 $\:\pm\:$0.01 ^i^	1.45 $\:\pm\:$0.01 ^g^	0.96 $\:\pm\:$0.07 ^g^	0.74 $\:\pm\:$0.10 ^f^
	3	0.86 $\:\pm\:$0.03 ^e^	0.39 $\:\pm\:$0.01 ^d^	0.45 $\:\pm\:$0.02 ^e^	0.11 $\:\pm\:$0.01 ^e^	2.23 $\:\pm\:$0.23 ^e^	1.34 $\:\pm\:$0.15 ^ef^	1.89 $\:\pm\:$0.02 ^g^	1.51 $\:\pm\:$0.02 ^e^	2.69 $\:\pm\:$0.12 ^c^	0.97 $\:\pm\:$0.07 ^d^
	5	1.22 $\:\pm\:$0.01 b	0.52 $\:\pm\:$0.01 ^a^	0.59 $\:\pm\:$0.03 ^c^	0.18 $\:\pm\:$0.00 ^b^	3.77 $\:\pm\:$0.15 ^d^	1.93 $\:\pm\:$0.12 ^c^	2.08 $\:\pm\:$0.02 ^e^	1.57 $\:\pm\:$0.04 ^d^	3.47 $\:\pm\:$0.04 ^a^	1.20 $\:\pm\:$0.04 ^c^
LSD		0.02	0.01	0.02	0.01	0.18	0.17	0.03	0.02	0.08	0.06

Values are mean ± SD of six determinations.

Values followed by the same letter, within the same column, were not significantly different (*p* > 0.05), according to Duncan’s multiple range test.

**Table 5:** Physicochemical properties of snacks with selected bee pollen.

Snacks	Level of addition [%]	Moisture [%]	Fat [%]	Expansion index	Bulk weight [g/L]	Texture [*N*]
Control	0	4.35 ± 0.05^ab^	27.79 $\:\pm\:$0.04 ^f^	2.83$\:\pm\:$0.38 ^a^	37.77 $\:\pm\:$1.77 ^d^	29.31$\:\pm\:$6.76 ^a^
RS	1	4.21 ± 0.01^b^	28.74 $\:\pm\:$0.10 ^c^	2.46 $\:\pm\:$0.31 ^bcd^	43.27 $\:\pm\:$1.70 ^b^	28.37 $\:\pm\:$6.62 ^ab^
	3	4.44 ± 0.02^a^	29.95 $\:\pm\:$0.04 ^a^	2.27 $\:\pm\:$0.30 ^def^	43.77 $\:\pm\:$1.58 ^b^	27.63 $\:\pm\:$6.97 ^abc^
	5	4.20 ± 0.08^b^	29.98 $\:\pm\:$0.21 ^a^	2.18 $\:\pm\:$0.38 ^ef^	46.76 $\:\pm\:$1.58 ^a^	27.42 $\:\pm\:$6.68 ^abc^
MF	1	4.13 ± 0.06^bc^	27.68 $\:\pm\:$0.22 ^f^	2.81 $\:\pm\:$0.34 ^a^	37.88 $\:\pm\:$0.61 ^d^	29.14 $\:\pm\:$5.70 ^ab^
	3	3.90 ± 0.07^d^	27.92 $\:\pm\:$0.22 ^ef^	2.60 $\:\pm\:$0.52 ^b^	39.56 $\:\pm\:$1.78 ^cd^	22.45 $\:\pm\:$6.58 ^de^
	5	3.80 ± 0.09^de^	28.40 $\:\pm\:$0.27 ^c^	2.53 $\:\pm\:$0.57 ^bc^	40.73 $\:\pm\:$1.65 ^c^	22.01 $\:\pm\:$6.64 ^de^
MP	1	4.22 ± 0.06^b^	27.90 $\:\pm\:$0.06 ^ef^	2.57 $\:\pm\:$0.31 ^bc^	37.56 $\:\pm\:$1.41 ^d^	24.14 $\:\pm\:$6.88 ^cd^
	3	4.24 ± 0.05^b^	28.16 $\:\pm\:$0.02 ^d^	2.11 $\:\pm\:$0.37 ^f^	43.61 $\:\pm\:$1.51 ^b^	22.05 $\:\pm\:$5.80 ^cd^
	5	4.02 ± 0.04^c^	28.52 $\:\pm\:$0.22 ^c^	2.06 $\:\pm\:$0.41 ^f^	44.17 $\:\pm\:$1.80 ^b^	18.47 $\:\pm\:$5.90 ^e^
PH	1	4.48 ± 0.05^a^	27.83 $\:\pm\:$0.01 ^f^	2.67 $\:\pm\:$0.49 ^ab^	39.13 $\:\pm\:$1.91 ^cd^	28.09 $\:\pm\:$5.85 ^abc^
	3	4.24 ± 0.04^b^	28.55 $\:\pm\:$0.16 ^c^	2.39 $\:\pm\:$0.39 ^cde^	40.49 $\:\pm\:$1.50 ^c^	27.31 $\:\pm\:$6.45 ^abc^
	5	3.84 ± 0.05^de^	29.31 $\:\pm\:$0.02 ^b^	2.28 $\:\pm\:$0.36 ^def^	42.90 $\:\pm\:$1.18 ^b^	25.45 $\:\pm\:$6.06 ^bcd^
LSD*		0.12	0.30	0.30	2.03	4.00

Values are mean ± SD of three determinations (moisture, fat,), ten determinations (expansion index), five determinations (bulk weight), twenty determinations (texture).

Values followed by the same letter, within the same column, were not significantly different (*p* > 0.05), according to Duncan’s multiple range test.

The snacks production process (extrusion and frying) reduced TPC and TFC in snacks compared to the mixtures from which they were produced. The smallest losses of TPC were observed in the control sample (< 5%) and the highest in the snacks with 5% additive of rapeseed bee pollen (65%). The losses of TFC ranged from 69% (control sample and snacks with 1% of multifloral bee pollen) to 80% (snacks with 3% of multifloral bee pollen). The antioxidant capacity of DPPH^•^ and FRAP also decreased (Table 4) after extrusion and frying, as expected as most of the functional compounds behind the antioxidant activity are thermosensitive. In general, the unit operations related to the snack preparation (extrusion and frying) resulted in significant reductions of the TPC and TFC, with the exception of the ABTS^•+^ in the control sample. The preservation of the biological activity of phenolics, during snacks production, above all depends on the resistance of individual compounds to the high temperature generated during these processes^47^. High temperature is considered to be the main degradation factor of phenolics^15^; however, some studies showed that frying may increase the antioxidant activity of ABTS^•+^, which was observed in the case of carrots and celery^48^ and in the case of snacks with the addition of dried, colored fleshed potatoes of the Herbie 26 variety^15^. The increase of antioxidant activity may be caused by melanoidins formed during the Maillard reaction, which have antioxidant potential. These compounds are formed from carbohydrates and amino acids at high temperature, e.g., during frying^15,49^.

Obtained snacks with the addition of bee pollens, regardless of the add-on level, were characterized by higher phenolics and flavonoids content as well as antioxidant capacity compared to the control sample. The highest level of TPC and antioxidant capacity ABTS^•+^ and FRAP determined in snacks were those reached after the 5% addition of multifloral and rapeseed bee pollens. In the study by Combrzyński et al.^12^, they proved that the content of phenolics and the antioxidant activity of potato snacks increased with the increase in the addition of cricket flour. Some authors emphasize that the antioxidant activity is influenced not only by the amount of phenolics, but also by the composition of this fraction, pH of the environment or even characteristic groups of specific compounds^40,50^.

### Physicochemical properties of snacks with selected bee pollen

The snacks obtained during the experiment contained from 3.80 to 4.48% of moisture, which indicates that the product was properly fried. Commercial snacks usually contain from 2.55 to 3.73% of water, and it is established that potato snacks should contain up to 4.5% moisture^51^. The addition of bee pollen reduced or did not significantly affect the moisture of the obtained products. Lower moisture was noticed in snacks enriched with 5% bee pollen (Table 5). High moisture content in snacks may deteriorate their quality and may become more elastic and rubberier; besides, it may also accelerate the process of fat decomposition, and the newly formed compounds may be toxic and have a negative impact on taste and smell^52^.

Fat content in obtained snacks ranged from 27.79% (control sample) to 29.98% (snacks with 5% add-on rape bee pollen). The addition of bee pollen caused an light increase in the fat content, although this increment was basically significant for the highest addition (5%). The highest level of fat content had snacks with addition of rapeseed bee pollen, which is linked with the high initial fat content of this type of bee pollen (12.76%). The fat content in snacks depends, among other factor, on the moisture of the pellets; a high moisture is normally related to an increased fat absorption. This is due to the fact that when frying snacks, the water evaporates and it is replaced by fat.

The degree of expansion of the obtained snacks ranged from 2.06 (snacks with 5% addition of multifloral with a large amount of *Papaver sp.*) to 2.83 (control sample). The expansion index decreased with the increase of bee pollen addition. Properly expanded potato snacks should have an expansion degree in the range 3 to 5^53^. In order to achieve the appropriate degree of expansion, pellets must have the appropriate moisture (approx. 12%), starch content (80–90%) and its degree of gelatinization (approx. 85%)^16^. The addition of bee pollen reduced the degree of expansion of obtained snacks, with the exception of 1% addition of multifloral and *Phacelia sp.* bee pollens, where the degree of expansion was statistically the same as in the control sample. With the increase in the level of bee pollen addition, regardless of the type of raw material used, the degree of expansion decreased. The lower values in the expansion index could have been caused by a reduction in the starch content in the recipe due to a higher proportion of bee pollen or/and an increase in the proportion of fat in the mixture (fat content in bee pollens ranged from 6.08 to 12.76%). The observed reduction in the expansion index may be attributed, at least in part, to the antifoaming properties of bee pollen. Kostić et al.^54^ reported that bee pollen suspensions did not exhibit foaming under the tested conditions. This lack of foaming capacity is likely related to the presence of surface-active lipids in bee pollen, which are known to destabilize foam. Lipids possess higher surface activity than proteins, allowing them to displace proteins from the air–water interface. This displacement reduces the thickness and cohesiveness of interfacial films, ultimately compromising foam stability. The addition of certain raw materials to the recipe may affect the degree of expansion of the snacks. The addition of flax and rapeseed pomace decreased, while the addition of modified starches increased, the degree of expansion of potato snacks^55^. The degree of expansion of extruded products may also be influenced by the sugar content in the mixture. Carvalho and Mitchell^56^, reports that increasing the sugar content may reduce the degree of expansion of the finished product. The bee pollen used contains significant amounts of sugar in its composition (Table 1), which may have significantly influenced the degree of expansion of the obtained snacks.

The bulk weight of the snacks was inversely proportional to the degree of expansion of the snacks. The calculated correlation coefficient was − 0.88. Snacks with a higher degree of expansion were characterized by a lower bulk density. Moreover, the bulk density increased with the increase of the additive. The lowest bulk weight was found in snacks with 5% addition of rapeseed bee pollen (46.76 g/L) and the smallest in snacks in the control sample (37.77 g/L), products with multifloral bee pollen (from 37.88 to 40.73 g/L) and with 1% addition of *Phacelia sp.* bee pollen (39.13 g/L). Market potato snacks had a bulk density of 43.75 to 66.71 g/L^51^. Low product moisture and/or high degree of expansion cause the product to have a low bulk weight^55^. The bulk weight also depends on the shape of the snacks. The regular form of snacks allows for a higher bulk density and, consequently, smaller unit packages^51,55^.

The force required to break the snacks obtained in the experiment ranged from 18.47 to 29.13 N. A relative relationship was also observed between the degree of expansion and the texture of the obtained products (correlation coefficient 0.5). The highest and lowest values of hardness were found in the control sample (29.13 N) and the snacks prepared using 5% of multifloral bee pollen with a predominance of *Papaver sp.* (18.47 N). In general, snacks with a high water content are less crispy, causing an increase in the force needed to break the snacks^51^; the products obtained in this study contained from 3.80 to 4.48% moisture. Optimal texture and expansion of snacks depend on precise control of moisture, temperature and frying time, as well as the proportions of ingredients such as proteins, starches and additives^57^. The addition of bee pollen (1%, 3%, 5%) reduced the force needed to break the snacks (Table 5), which means that these snacks had a softer texture. It can be caused by the thickness of the snacks; in fact, products with the addition of bee pollen were thinner than the control samples.

The color of bee pollen influenced on the color of snacks obtained with their participation. The addition of bee pollen caused that the obtained snacks were characterized by a darker color with a greater proportion of red color (Table 6). Snacks with *Phacelia sp.* bee pollen were dark beige, with rapeseed and multifloral yellow, and with multifloral with strong predominance of *Papaver sp.*, yellow-beige, and the control sample was light-yellow. The addition of bee pollen affected the color of potato snacks, even in the case of a slight enrichment. The smallest difference in color compared to the control sample was observed in snacks with 1% of rapeseed bee pollen (ΔE = 1.53). Such a difference in color is noticeable only by an experienced observer. With the increase in the addition of bee pollen, a higher color change was observed compared to the color of the sample without the addition of bee pollen. Snacks with 5% of bee pollen, and snacks with 3% of *Phacelia sp.* and multifloral bee pollen with a strong predominance of *Papaver sp*., were characterized by a different color than snacks of the control sample (ΔE in the range from 5.17 to 12.44). During frying, Maillard reactions occur, in which colored compounds are formed. Furthermore, the overall color changes could be due to a number of factors such as pigment degradation, pigment polymerisation, reactions with other components of the formulation, non-enzymatic browning, oxidation of tannins, and other reactions completely unrelated to the added raw material^58^.

**Table 6:** Color coordinates of snacks with selected bee pollen.

Snack	Level of adition [%]	Colour parameters					ΔE
		L*	a*	b*	C	h	
Control	0	75.29 $\:\pm\:$0.12 ^a^	3.18 $\:\pm\:$0.03 ^k^	24.39 $\:\pm\:$0.07 ^i^	24.60 $\:\pm\:$0.08 ^h^	82.57 $\:\pm\:$0.05 ^a^	-
RS	1	73.89 $\:\pm\:$0.18 ^b^	3.75 $\:\pm\:$0.15 ^j^	26.58 $\:\pm\:$0.34 ^g^	26.84 $\:\pm\:$0.36 ^f^	81.98 $\:\pm\:$0.21 ^b^	1.53
	3	71.06 $\:\pm\:$0.20 ^e^	4.70 $\:\pm\:$0.14 ^h^	29.00 $\:\pm\:$0.12 ^c^	29.38 $\:\pm\:$0.14 ^c^	80.80 $\:\pm\:$0.24 ^c^	3.50
	5	68.06 $\:\pm\:$0.21 ^i^	6.07 $\:\pm\:$0,12 ^c^	30.12 $\:\pm\:$0.24 ^a^	30.72 $\:\pm\:$0.26 ^a^	78.61 $\:\pm\:$0.16 ^h^	7.30
MF	1	72.72 $\:\pm\:$0.06 ^c^	4.74 $\:\pm\:$0.02 ^h^	26.99 $\:\pm\:$0.10 ^f^	27.41 $\:\pm\:$0.10 ^e^	80.03 $\:\pm\:$0.03 ^e^	1.99
	3	71.93 $\:\pm\:$0.25 ^d^	4.87 $\:\pm\:$0.08 ^g^	28.63 $\:\pm\:$0.13 ^d^	29.04 $\:\pm\:$0.14 ^d^	80.35 $\:\pm\:$0.13 ^d^	2.24
	5	70.01 $\:\pm\:$0.14 ^f^	5.85 $\:\pm\:$0.06 ^d^	29.92 $\:\pm\:$0.20 ^a^	30.49 $\:\pm\:$0.21 ^b^	78.93 $\:\pm\:$0.11 ^g^	5.17
MP	1	69.01 $\:\pm\:$0.17 ^h^	5.39 $\:\pm\:$0.05 ^f^	25.78 $\:\pm\:$0.06 ^h^	26.34 $\:\pm\:$0.05 ^g^	78.19 $\:\pm\:$0.13 ^i^	4.64
	3	65.33 $\:\pm\:$0.10 ^j^	7.04 $\:\pm\:$0.07 ^b^	28.25 $\:\pm\:$0.19 ^e^	29.12 $\:\pm\:$0.20 ^d^	76.01 $\:\pm\:$0.05 ^j^	7.86
	5	63.53 $\:\pm\:$0.13 ^k^	7.89 $\:\pm\:$0.09 ^a^	29.33 $\:\pm\:$0.08 ^b^	30.37 $\:\pm\:$0.09 ^b^	74.95 $\:\pm\:$0.13 ^k^	8.38
PH	1	69.51 $\:\pm\:$0.14 ^g^	4.40 $\:\pm\:$0.09 ^i^	23.86 $\:\pm\:$0.27 ^j^	24.26 $\:\pm\:$0.28 ^i^	79.56 $\:\pm\:$0.12 ^f^	2.23
	3	63.25 $\:\pm\:$0.10 ^l^	5.61 $\:\pm\:$0.05 ^e^	20.81 $\:\pm\:$0.10 ^k^	21.55 $\:\pm\:$0.11 ^j^	74.91 $\:\pm\:$0.09 ^k^	9.50
	5	60.60 $\:\pm\:$0.12 ^ł^	6.16 $\:\pm\:$0.03 ^c^	19.33 $\:\pm\:$0.09 ^l^	20.29 $\:\pm\:$0.09 ^k^	72.32 $\:\pm\:$0.08 ^l^	12.44
LSD*		0.20	0,10	0.20	0.21	0.15	-

Values are mean ± SD of five determinations.

Values followed by the same letter, within the same column, were not significantly different (*p* > 0.05), according to Duncan’s multiple range test.

### Sensory analysis of snacks

**Fig. 3:**
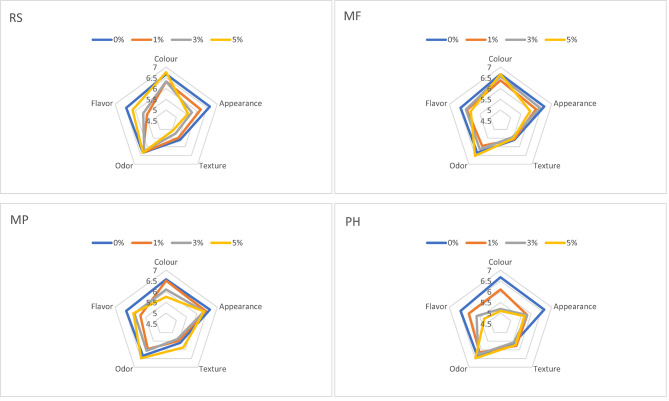
Sensory profiles of snacks prepared using selected bee pollen.

The snacks of the control sample and those with 5% add-on rape and multifloral bee pollens were rated the highest in terms of color of products. The worst notes in terms of color were snacks with 3 and 5% of *Phacelia sp.* and multifloral bee pollen with a predominance of *Papaver sp.* bee pollen. Probably it is connected with it that snacks with rape and multifloral as well as control sample were yellow and with *Phacelia sp*. and multifloral with predominance of *Papaver sp*. were in the shadow of beige (Table 5) and the color of snacks with multifloral with predominance of *Papaver sp.* was less saturated than another samples (Fig. 3).

Color of snacks refers to the visual appearance or pigmentation of snack foods, which can be influenced by natural ingredients or preparation methods (like baking, frying, or roasting). The color of a snack often affects its appeal, flavor expectations, and even perceived freshness or healthiness.

High quality potato snacks are characterized by fully expanded products with a soft surface, crunchy and hard. Snacks fulfilling these requirements were found in samples prepared using multifloral and *Phacelia sp.* bee pollens, which texture and structure was quite similar to that of the control samples.

The addition of bee pollen should provide potato snacks with floral and honey odor and sweet flavor; however, it can also provide excessive bitterness, depending on the type of flowers which have originated the bee pollen^59,60^. In this sense, the phacelia bee pollen provided negative aromas and a bitter taste, which reduced the scores of the odor and flavor attributes. On the other hand, the rapeseed bee pollen provided a sweet taste which increase the score of the flavor attribute.

### Principal components analysis (PCA)

**Fig. 4:**
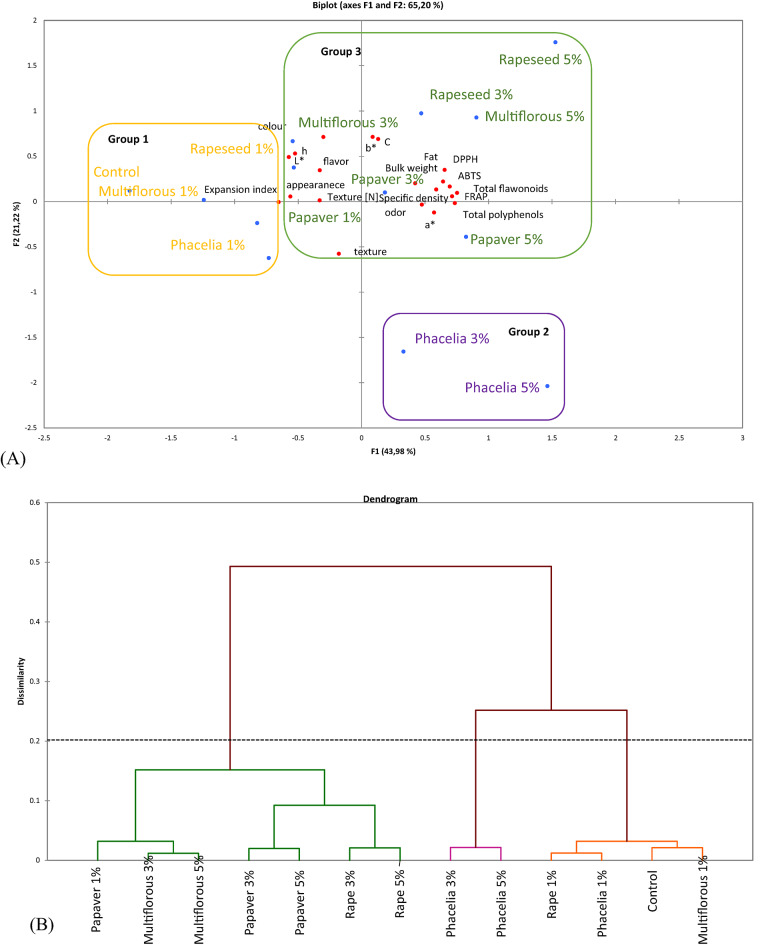
(**A**) Principal components analysis (PCA) and (**B**) cluster map for snacks with 1, 3 and 5% addition of bee pollens.

PCA was conducted to summarize the appropriate grouping of all physicochemical parameters and sensory attributes linked to the type of bee pollen and level of its addition to the recipe of snacks (Fig. 4A). The first two main components explained 65.20% of the total variance (PC1 = 43.98% and PC2 = 21.22%). The first principal component was responsible for the differences between the color parameters (a*, b*,* C*), flavor, antioxidant activity (ABTS^•+^, DPPH^•^, FRAP), TPC and TFC as well as fat content, bulk weight and specific density. In turn, PC2 was responsible for the differences in most of the sensory attributes (flavor, structure, texture, color) and also instrumental texture, color parameters (L*,* h°*) as well as expansion index.

In addition, PCA showed differences as affected by the kind of bee pollen and level of addition. The TPC in the snacks correlated positively with antioxidant activity (ABTS^•+^, DPPH^•^, FRAP) and TFC as well as with color parameter a* and some sensory attributes (structure and flavor). Moreover, TPC was negatively correlated with color parameter L* and structure. In addition, fat content was positively correlated with TFC, bulk weight, specific density and sensory attribute structure. The expansion index was negatively correlated with TFC, bulk weight, specific density and color parameter b*. On the dendrogram presented in Fig. 4B **it** can be observed how the samples are grouped into 3 main groups:

- *Group 1*: snacks of control sample, with 1% addition of bee pollens (MF, RS and PH);
- *Group 2*: snacks with 3% and 5% addition of *Phacelia sp*. bee pollen;
- *Group 3*: snacks with 1% and 3% *Papaver sp.* bee pollen, 3% and 5% of rapeseed bee pollen as well as 3% and 5% of multifloral bee pollen.

## Conclusions

The bee pollen used in the research varied in terms of basic chemical composition, total polyphenol and flavonoid content, as well as antioxidant activity and color. Rapeseed bee pollen had the highest TPC, TFC and the highest DPPH^•^ and FRAP antioxidant activity, while the phacelia bee pollen showed the highest antioxidant activity in the ABTS^•+^ test. The snacks production process (extrusion and frying) resulted in significant decreases, as compared to the ingredients mixture (dough), for the content most of the parameters studied, including for instance, the TPC (from 4.76 to 65%), the TFC (from 69 to 80%), and the antioxidant activity (DPPH^•^, from 9 to 37%; FRAP, from 13 to 68%). The physicochemical properties, including the color of the obtained snacks, depended on the type and degree of addition of bee pollen used. The use of bee pollen as additive in the production of fried potato snacks contributed to obtaining products with an increased TPC ranging from 1.3 to 2.6 and TFC from 2.0 to 4.8 times compared to snacks without the addition of bee pollen. Besides, enriching the snacks mixes with bee pollen increased the antioxidant activity of the resulting products. The TPC, TFC and the antioxidant activity of snacks enriched with bee pollen depended on the type of bee pollen used, the properties of the phenolics contained in it and the degree of its addition, with the best results being obtained after the addition of 3% multifloral bee pollen resulted in potato snacks fulfilling the requirements to be considered as high-quality snacks. Potato snacks supplemented with bee pollen could be classified as a new product in the fortified food market. Bee pollen is a good source of phenolics, but when consumed directly it may have a bitter and astringent taste. The research carried out may encourage producers to increase the offer of healthy snacks, and researchers to increase research on enriching food with bee pollen.

## Electronic supplementary material

Below is the link to the electronic supplementary material.

Supplementary Material 1

## Data Availability

The datasets used and/or analysed during the current study available from the corresponding author on reasonable request.
