# Supplementary material for: Bee pollen as a source of phenolic compounds in potato snacks
Source: Sci Rep. 2025 Jul 7;15:24203. doi: 10.1038/s41598-025-09776-4 (PMC12234791; doi:10.1038/s41598-025-09776-4)
Supplement: Supplementary file 1 — Supplementary Material 1 [file 41598_2025_9776_MOESM1_ESM.docx]

| **Attribute** | **Point** | **Requirement** | **Sample** | | | | | | |
| --- | --- | --- | --- | --- | --- | --- | --- | --- | --- |
|  |  |  | **125** | **258** | **963** | **136** | **589** | **247** | **359** |
| **Color** | **7** | Uniform, creamy-yellow / cream / beige / typical for the ingredients used |  |  |  |  |  |  |  |
|  | **6** | Creamy-white / gray-cream / gray-beige |  |  |  |  |  |  |  |
|  | **5** | Gray-cream, few dark spots |  |  |  |  |  |  |  |
|  | **4** | Non-uniform, typical for the ingredients used |  |  |  |  |  |  |  |
|  | **3** | Non-uniform, yellow-brown |  |  |  |  |  |  |  |
|  | **2** | Non-uniform, brown-yellow |  |  |  |  |  |  |  |
|  | **1** | Brown, burnt product |  |  |  |  |  |  |  |
| **Appearance** | **7** | Uniform expansion, homogeneous porous structure, gently wavy |  |  |  |  |  |  |  |
|  | **6** | Expanded, homogeneous structure, wavy |  |  |  |  |  |  |  |
|  | **5** | Expanded, structure with uneven porosity, irregularly wavy |  |  |  |  |  |  |  |
|  | **4** | Partially unexpanded, non-uniform structure, twisted |  |  |  |  |  |  |  |
|  | **3** | Partially expanded product |  |  |  |  |  |  |  |
|  | **2** | Slight expansion, very fine porosity |  |  |  |  |  |  |  |
|  | **1** | Unexpanded product, dense non-porous structure |  |  |  |  |  |  |  |
| **Texture** | **7** | Delicate, crispy, crumbling |  |  |  |  |  |  |  |
|  | **6** | Crispy, high breakability |  |  |  |  |  |  |  |
|  | **5** | Crispy, detectable rubbery spots, brittle |  |  |  |  |  |  |  |
|  | **4** | Crispy, detectable hard spots, brittle |  |  |  |  |  |  |  |
|  | **3** | Not crispy, medium breakability |  |  |  |  |  |  |  |
|  | **2** | Hard, grainy, low breakability |  |  |  |  |  |  |  |
|  | **1** | Very hard, unbreakable |  |  |  |  |  |  |  |
| **Odor** | **7** | Typical potato, slightly fried fat |  |  |  |  |  |  |  |
|  | **6** | Potato, fried potato dough |  |  |  |  |  |  |  |
|  | **5** | Potato with a distinct fat note |  |  |  |  |  |  |  |
|  | **4** | Fatty, with a noticeable foreign smell |  |  |  |  |  |  |  |
|  | **3** | Atypical, burnt |  |  |  |  |  |  |  |
|  | **2** | Atypical, fatty, burnt |  |  |  |  |  |  |  |
|  | **1** | Atypical, burnt, bitter, rancid fat |  |  |  |  |  |  |  |
| **Flavor** | **7** | Delicate, potato-like with a hint of fried fat |  |  |  |  |  |  |  |
|  | **6** | Characteristic of fried potato dough |  |  |  |  |  |  |  |
|  | **5** | Potato-like, with a taste of the used oil |  |  |  |  |  |  |  |
|  | **4** | Potato-like, with a foreign taste, burnt |  |  |  |  |  |  |  |
|  | **3** | Atypical, burnt |  |  |  |  |  |  |  |
|  | **2** | Atypical, bitter, burnt |  |  |  |  |  |  |  |
|  | **1** | Atypical, burnt, bitter, rancid fat |  |  |  |  |  |  |  |

Comments:
